# Supplementary material for: Cloud BioLinux: pre-configured and on-demand bioinformatics computing for the genomics community
Source: BMC Bioinformatics. 2012 Mar 19;13:42. doi: 10.1186/1471-2105-13-42 (PMC3372431; doi:10.1186/1471-2105-13-42)
Supplement: Additional file 1 — Supplementary 1 Cloud BioLinux software documentation in the form of a mini, self-contained website. Users need to download and uncompress the .zip file, and open through a web browser the "index.html" file available on the main directory. (ZIP 1823 kb). [file 1471-2105-13-42-S1.ZIP › Cloud-BioLinux-Package-Documentation/docs/pfmake.html]

Bio-Linux Software Documentation Pages

Back to search form

## pfmake

|  |  |
| --- | --- |
| Name | pfmake |
| Description | **pfmake** is part of the pftools package, a collection of tools making use of generalised profiles for sequence analysis.  **pfmake** generates a PROSITE profile from a multiple sequence alignment using methods described by Gribskov *et* *al*.(1990), Luethy*et* *al*. (1994), and Thompson *et* *al*. (1994), with modifications to exploit the features of the new profile format.  **References:**  Bucher P, Karplus K, Moeri N & Hofmann, K. (1996). A flexible motif search technique based on generalized profiles. Comput. Chem. 20:3-24.    Gribskov M, Luethy R & Eisenberg D (1990). Profile analysis. Meth. Enzymol. 183:146-159.    Luethy R, Xenarios I & Bucher P (1994). Improving the sensitivity of the sequence profile method. Prot. Sci.3:139-146.    Thompson JD, Higgins DG & Gibson TJ (1994) Improved sensitivity of profile searches through the use of sequence weights and gap excision. Comput. Appl. Biosci. 10:19-29. |
| Homepage | http://www.isrec.isb-sib.ch/profile/profile.html |
| Remote Documentation | http://www.isrec.isb-sib.ch/ftp-server/pftools/pft2.3/documentation/pfmake.1.html |
